# *Clostridium difficile* Toxins A and B: Insights into Pathogenic Properties and Extraintestinal Effects

**DOI:** 10.3390/toxins8050134

**Published:** 2016-05-03

**Authors:** Stefano Di Bella, Paolo Ascenzi, Steven Siarakas, Nicola Petrosillo, Alessandra di Masi

**Affiliations:** 1 2nd Infectious Diseases Division, National Institute for Infectious Diseases “L. Spallanzani”, Rome 00149, Italy; nicola.petrosillo@inmi.it; 2 Department of Science, Roma Tre University, Rome 00154, Italy; paolo.ascenzi@uniroma3.it (P.A.); alessandra.dimasi@uniroma3.it (A.M.); 3 Department of Microbiology and Infectious Diseases, Concord Repatriation General Hospital, Sydney 2139, Australia; steven.siarakas@sswahs.nsw.gov.au

**Keywords:** *Clostridium difficile*, toxins, pathogenesis

## Abstract

*Clostridium difficile* infection (CDI) has significant clinical impact especially on the elderly and/or immunocompromised patients. The pathogenicity of *Clostridium difficile* is mainly mediated by two exotoxins: toxin A (TcdA) and toxin B (TcdB). These toxins primarily disrupt the cytoskeletal structure and the tight junctions of target cells causing cell rounding and ultimately cell death. Detectable *C. difficile* toxemia is strongly associated with fulminant disease. However, besides the well-known intestinal damage, recent animal and *in vitro* studies have suggested a more far-reaching role for these toxins activity including cardiac, renal, and neurologic impairment. The creation of *C. difficile* strains with mutations in the genes encoding toxin A and B indicate that toxin B plays a major role in overall CDI pathogenesis. Novel insights, such as the role of a regulator protein (TcdE) on toxin production and binding interactions between albumin and *C. difficile* toxins, have recently been discovered and will be described. Our review focuses on the toxin-mediated pathogenic processes of CDI with an emphasis on recent studies.

## 1. Introduction

*Clostridium difficile* is a Gram-positive, anaerobic, spore-forming, toxin-producing bacillus. *C. difficile* is the most common cause of nosocomial infections in the United States, surpassing methicillin resistant *Staphylococcus aureus* [1]. A total of 15%–25% of all cases of antibiotic-associated diarrhea result from *Clostridium difficile* infection (CDI) [2]. Its incidence and associated mortality are progressively increasing in Western countries including a number of well categorized outbreaks in the US, Canada, and Europe [3].

*C. difficile* can change into its vegetative disease causing state when it reaches the intestine of humans. This anaerobic bacterium is well suited to the anaerobic environment of the colon, and the presence of glycine and cholate derivatives facilitates the germination of *C. difficile* spores. In healthy intestines with a normal microbiota, cholate derivatives are further processed by other bacteria preventing germination of *C. difficile* spores. Patients receiving broad-spectrum antibiotic treatment have much of their intestinal microflora disrupted or killed, preventing the metabolism of cholate which in turn facilitates *C. difficile* germination and overgrowth. The distraction of host microbes by the antibiotic treatment gives *C. difficile* more space in the intestine [4].

Besides antibiotic consumption, the main risk factors associated with the development of CDI are advanced age, impairment in humoral immunity, renal disease, hypoalbuminemia [5,6,7,8]. The clinical manifestations associated with CDI range from asymptomatic colonization and mild diarrhea to toxic megacolon and life-threatening fulminant colitis [9]. The pathogenetic effects of *C. difficile* are mainly secondary to the production of two exotoxins: toxin A (TcdA) and toxin B (TcdB) [9].

During the last 20 years, the scientific community interest toward *C. difficile* has significantly increased. The increasing focus on this bacterium is due to the burden of a disease that has significantly increased in terms of incidence and mortality, alongside with the parallel increase of the disease recurrences. To make matters worse, in a growing portion of cases, antibiotic therapy alone is no longer able to control the infection, and new therapeutic approaches such as fecal transplantation have eradicated *C. difficile* colonization and infection [10,11].

The global spread of the highly sporulating *C. difficile* ribotype BI/NAP1/027 has changed traditional epidemiology. Several factors have contributed to the increased virulence pattern of this ribotype. Importantly, it has been shown that this strain is unable to downregulate toxin production, with consequent high levels of toxin synthesis. *In vitro* studies with ribotype BI/NAP1/027 strains demonstrate that these isolates produce 16- and 23-fold higher levels of TcdA and TcdB, respectively, and since toxin production is closely related to spore production, ribotype BI/NAP1/027 produces greater numbers of spores compared to other ribotypes. This increased toxin and spore production capability helps ribotype BI/NAP1/027 to compete and become the dominant strain within any environment to which it is introduced [12]. Further, this increased toxin capability is closely linked to the pathogenesis of CDI by its ability to inactivate Rho GTPases expressed in host cells causing several direct and indirect cytopathic effects, ultimately leading to colonocyte death, loss of intestinal barrier function, and neutrophilic colitis [13]. Recently, a *C. difficile* NAP1/027 strain from the hypervirulent clade 2 carrying a variant TcdB (TcdB_NAP1V_) has been identified [14]. In contrast to the TcdB expressed from the classic NAP1/027 strain, TcdB_NAP1V_ does not glucosylate Rho and partially targets Cdc42. Furthermore, it induces a significantly lower quantity of pro-inflammatory mediators, thus highlighting that the specific small GTPases glucosylated by *C. difficile* toxins are crucial in determining the biological outcome of the pathogen [14].

In this review, we provide an updated overview of the structure and function of TcdA and TcdB, as well as of their cellular and systemic effects in the host cells.

## 2. *Clostridium difficile* Toxins

Most *C. difficile* strains produce two major toxins, *i.e.*, TcdA and TcdB, generated by the genes *tcd*A and *tcd*B within the organism’s Pathogenicity loci (PaLoc), while certain *C. difficile* strains may produce a binary toxin called *C. difficile* transferase (CDT), closely related to the *Clostridium perfringens* binary toxin. All these toxins are part of the large clostridial glucosylating toxin (LCGT) family, more appropriately called clostridial glucosylating toxins [15,16]. Additionally, the lethal and hemorrhagic toxins from *C. sordelli*, α-toxin from *C. novyi*, and the large cytotoxin from *C. perfrigens* belong to this family [17,18].

There are only a few strains of *C. difficile* that can synthesize CDT in the absence of TcdA and TcdB, and the role of CDT in *C. difficile* infection is still unclear. Although, strains only producing CDT have been isolated from patients with colitis supporting the hypothesis that CDT is involved in the pathogenesis process. However, the incidence of *C. difficile* infections related to strains only producing CDT is low, and the symptoms are moderate. In addition, these strains yield no severe lesions of enteritis in experimental animal models [15,19,20].

*C. difficile* strains show a great genetic variability and are divided into multiple toxin-types based on genetic variations of *tcdA* and *tcdB*, encoding for TcdA and TcdB, respectively [21].

### 2.1. Regulation of Expression and Secretion of TcdA and TcdB

**Figure 1:**
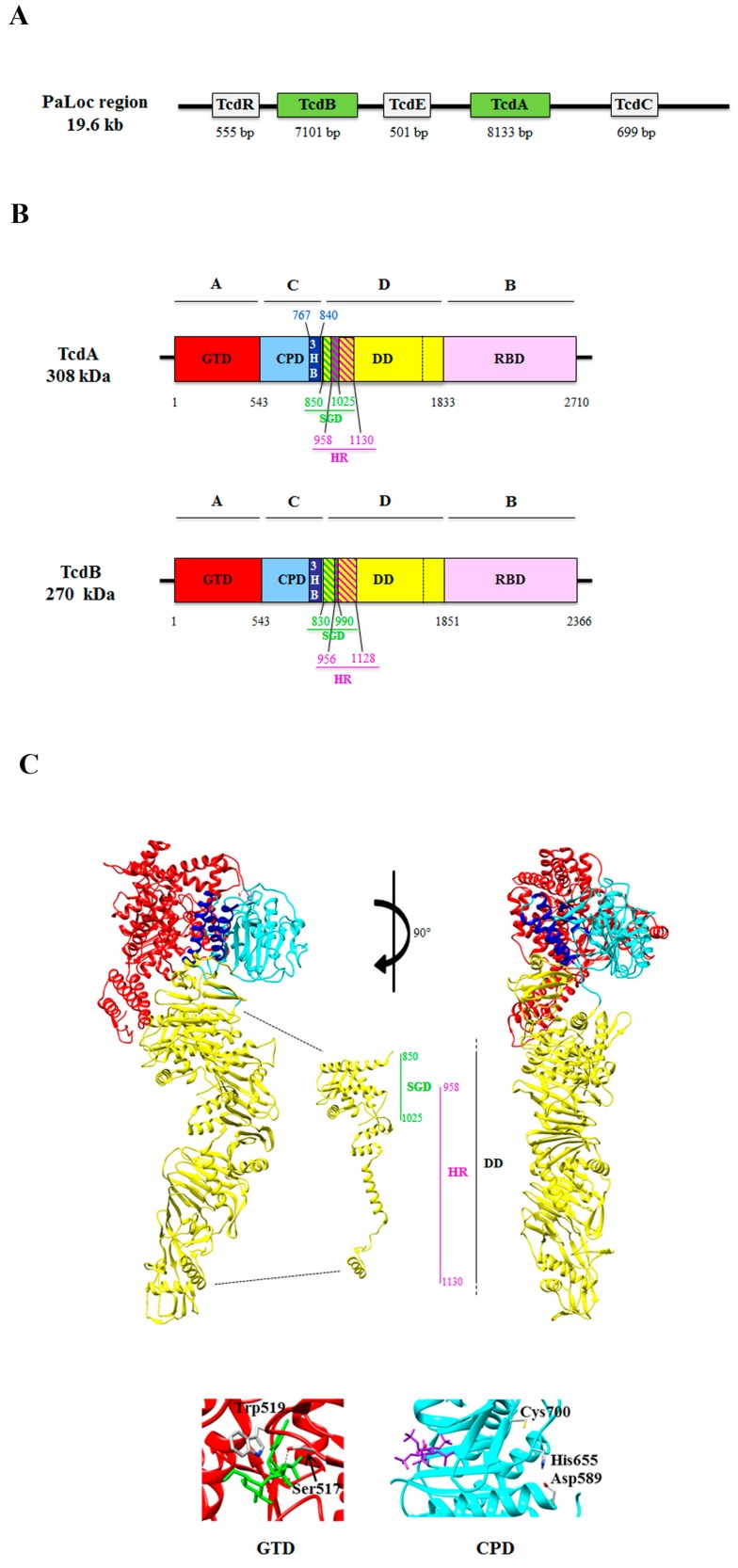
(**A**) Schematic representation of the PaLoc region containing the following genes: *tcdR*, *tcdB*, *tcdE*, *tcdC*, and *tcdA*. Below each gene, the number of base pairs (bp) is indicated. (**B**) Schematic representation of the multi-modular domain structure described as the ABCD model (A: biological activity; B: binding; C: cutting; D: delivery) domain structure of TcdA and TcdB. The two toxins are composed of: the A domain, corresponding to the *N*-terminal glucosyltransferase domain (GTD) (**red**) [32,33]; the C domain, corresponding to the cysteine protease domain (CPD) (**cyan**) and the three-helix bundle domain (3HB) (**blue**) identified in TcdA [34], and possibly present also in TcdB; the D domain, which corresponds to the delivery hydrophobic domain (DD) (**yellow**), containing the small globular domain (SGD) in TcdA (**green** and **yellow** diagonal lines) [34] that corresponds to the minimal pore forming region (MPFR) in TcdB (**green** and **yellow** diagonal lines) [35], and overlapping only partially (**purple** and **green** diagonal lines) to the hydrophobic region (HR) of TcdA [36] and TcdB [35] (**purple** and **yellow** diagonal lines); the B domain, corresponding to the receptor binding domain (RBD) (**pink**) [34,37]. (**C**) **Top**: two three-dimensional structures of TcdA are shown, rotated 90° each other (**red**: GTD; **cyan**: CPD; **blue**: 3HB; **yellow**: DD; **pink**: RBD), with a detail of the *N*-terminal region of the D domain showing the SGD and HR; right bottom: detail of the loop containing the amino acid residues 516–522 of the GTD, in which the conserved Ser517 residue forms a hydrogen bond with the phosphate group present in the UDP-glucose, and the conserved Trp519 forms a hydrogen bond with the glycosidic oxygen (PDB: 3SRZ; [18]); left bottom: detail of the catalytic triad Asp589-Hys655-Cys700 of the CPD, which is responsible for the proteolytic cleavage of the toxin dependent upon inositol hexakisphosphate (Insp6) (**purple**) binding (PDB: 3HO6; [38]). The three-dimensional structures were drawn with the UCSF-Chimera package [39]. For details, see text.

The *tcdA* and *tcdB* genes are located on the pathogenicity locus (PaLoc), a 19.6 kb chromosomally integrated DNA sequence [22,23]. The PaLoc also contains three other genes: (i) *tcdR*, encoding an alternative RNA polymerase sigma factor that is responsible for *tcdA* and *tcdB* expression [24]; (ii) *tcdE*, encoding a putative holin necessary for the extracellular release of both toxins [25,26,27,28]; and (iii) *tcdC*, which negatively regulates TcdA and TcdB synthesis [23,29,30,31] (Figure 1A).

PaLoc can be horizontally transferred to non-pathogenic strains characterized by the lack of *tcdA* and *tcdB*, converting them in pathogenic strains producer [21,40,41,42]. Although PaLoc possesses some characteristics of a mobile genetic element, it does not appear to be intrinsically mobile and is located at the same site in all toxigenic *C. difficile* strains [21].

The expression of TcdA and TcdB is dependent upon environmental conditions and global regulators, including the availability of specific nutrients, temperature changes, and alteration of the redox potential [43,44,45,46]. Usually, the toxin genes are transcribed when bacteria enter the stationary phase in which nutrient limitation or accumulation of growth inhibiting substances occurs [23,47]. In addition, the carbon sources [47,48] or specific amino acids [49,50] inhibit toxin gene expression. The complex and environmentally dependent manner in which *C. difficile* controls the expression of TcdA and TcdB clearly indicates that the bacteria’s nutritional status strongly influences their ability to cause damage to the host cells. The use by *C. difficile* of global metabolic regulators, such as CcpA (a protein of the Lac repressor family) [51], CodY (a global regulator involved in the adaptive response to nutrient limitation in Gram-positive bacteria) [52], and Rex (a redox-dependent transcriptional repressor that plays a key role in regulating anaerobic metabolism) [53] implies that virulence is a mechanism for improving nutrient availability [46].

Once expression of the toxin genes is induced, the toxin proteins accumulate inside the cell and are slowly released over the course of several hours. In particular, upon reaching the intestine, *C. difficile* enters a vegetative state, begins to spread, and starts to secrete TcdA and TcdB [13].

### 2.2. Structure of TcdA and TcdB

TcdA and TcdB share a common domain structure with 44% sequence identity and approximately 66% sequence similarity, the greatest diversity in sequence being confined to their *C*-terminal binding domains. The homology between the two toxins and the similar modes of entry in the host cell suggest that these toxins adopt similar 3D structures. Indeed, both toxins have a bilobe globular “head” domain (corresponding to the delivery domain), a long tail domain (corresponding to the receptor-binding domain), and a short inner tail domain (that contains the glucosyltransferase domain) [54,55].

Overall, the two toxins have similar enzymatic activities [55,56] and share a multi-modular domain structure described as the ABCD model (A: biological activity; B: binding; C: cutting; D: delivery) [57] (Figure 1B) [32,33,34,37]. Region A (*i.e.*, the short tail region) is located at the *N*-terminus and contains the 63 kDa glucosyltransferase domain (GTD) that acts on the small GTPases involved in regulation of the cytoskeleton [33,58,59]; region B (*i.e.*, the long tail region), placed at the *C*-terminal, is involved in receptor binding [36] and consists of combined repeated oligopeptides (CROPs) that form the receptor binding domain (RBD) [57]; region C corresponds to the cysteine protease domain (CPD) [60] that promotes the auto-catalytic cleavage of the toxins; and region D (*i.e.*, the head domain, also called the delivery domain (DD)) is involved in the translocation of the toxins into the cytosol [35], as well as their binding to target cells [35,59,61].

#### 2.2.1. The GTD Domain (A Domain)

The GTD is responsible for transferring a glucose from the UDP-glucose to the switch I region of Rho-family GTPases and its structure is conserved among the GTDs present in several large glucosylating toxins.

The GTD crystal structures of TcdA have been determined in the presence and absence of the UDP-glucose at 2.6 Å (PDB: 3SRZ) and 2.2 Å (PDB: 3SS1) resolution, respectively. The molecule is composed of a core GT-A fold surrounded by multiple helical projections [62]: The *N*-terminal projection seems to act as a membrane localization domain (MLD) targeting the GTD to the site of membrane-bound GTPases [63,64], whereas the other projections are thought to contribute to the GTPase substrate specificity (Figure 1C) [34,38,39,65,66]. The comparison of the apo- and UDP-glucose-bound structures shows a significant difference in the position of the 516–522 loop. This loop contains a conserved serine, *i.e.*, Ser517 in TcdA, which forms a hydrogen bond with the phosphate group present in the UDP-glucose, and a conserved tryptophan, *i.e.*, Trp519 in TcdA, which forms a hydrogen bond with the glycosidic oxygen. In the apo structure, the loop is shifted so that the Trp519 residue is located ~10 Å away from its position in the UDP-glucose-bound structure [65]. While the UDP-glucose is intact in the TcdA-GTD crystal structure, the UDP-glucose is hydrolyzed in the TcdB-GTD crystal structure. This is consistent with the observation that TcdB has a higher rate of UDP-glucose hydrolysis [67,68] than TcdA, although the enzymatic core of the two proteins appear highly similar [18]. Indeed, the residues and waters involved in the UDP-binding and catalysis are conserved, and the binding of UDP-glucose is nearly identical [18].

Despite the conserved core structures of the TcdA and TcdB GTDs, a high divergence in the surface residues has been reported, particularly in the GTPase-binding surface adjacent to the UDP-glucose site [18,69]. In particular, the amino acid changes in this region alter the electrostatic properties of the enzymes surface, suggesting that the toxins GTD may have different substrate specificities within the host cell. A further aspect that could further differentiate the toxins activities in cells is represented by the differences in the electrostatic potential properties of the MLD region. Indeed, the TcdB MLD is markedly more charged than that of TcdA, the “front” surface being dominated by a highly basic patch, whereas the opposite face being almost entirely acidic. Furthermore, TcdA has a smaller basic area on the front of the MLD and lacks the negatively charged patch on the back [65].

Notably, even though for a long time TcdB was believed to possess a glucosyltranferase activity 100–1000 times more potent than TcdA [70], recently, the determination of TcdA and TcdB glucosyltranferase activity against some Rho family members, both *in vitro* and in cells, highlighted that TcdA also possesses an enzymatic activity similar to that of TcdB, being able to modify several substrates (*i.e.*, Cdc42, RhoA, and Rap2A) [18]. Such discrepancy has been ascribed to the fact that previous studies used the holotoxins [70], in which the glucosyltranferase activity is reduced but is restored after the autoproteolytic activation and the GTD release [65]. Since TcdB undergoes autoprocessing much more readily than TcdA [71], the ~1000-fold difference in the cytopathic potency between TcdA and TcdB is not due to differences in the glucosyltranferase activity but rather in differences in the binding and/or delivery of the GTD into the target cell [18]. Of note, the NAP1V/RT019 hypervirulent strain recently isolated [14] expresses a variant form of TcdB, as demonstrated by the different macrorestriction pattern of this toxin compared to the one produced by the classical NAP1/027 strain. In particular, the main differences between the two TcdB variants reside within the *N*-terminal region. A detailed analysis and comparison of the sequences from the different TcdB toxins indicated that the primary sequence of the GTD present in TcdB_NAP1V_ is more closely related to the *C. sordellii* lethal toxin (TcsL) and *C. difficile* variant TcdB proteins [72,73]. Indeed, the identity in the first 546 amino acid residues between TcdB_NAP1V_ and TcdB_NAP1_ is 80%, whereas the identity in the substrate specificity domain (region comprised between the amino acid residues 365 and 516) is only 62%. Although the core residues of the GTDs are conserved, the surface residues predicted to be involved in the substrate recognition are divergent. This is consistent with the observation that RhoA was detected only in lysates prepared from cells intoxicated with TcdB_NAP1V_, suggesting that the ability to target this small GTPase is the main difference at the level of substrates between the two toxins [14]. Although TcdB_NAP1V_ and TcdB_NAP1_ showed similar cytopathic potencies, TcdB_NAP1_ was able to induce a higher release of TFN-α, a higher myeloperoxidase (MPO) activity, and higher levels of IL-1β and IL-6 [14].

Overall, the enzymatic activity of TcdA may have important implications in the pathogenesis of *C. difficile*. Since Rap proteins are known to be important in the regulation of cell-cell junctions, their inactivation by TcdA could be important for the disruption of the intestinal epithelium integrity. Of note, other Ras family members are involved in the pathways that regulate cell proliferation and survival [74]; therefore, the modification of Ras substrates by TcdA represents a key process in *C. difficile*-associated disease [65,72,75].

#### 2.2.2. The Receptor Binding Domain (B Domain)

The B domain, located at the *C*-terminus of *C. difficile* toxins, interacts with carbohydrate structures, such as Gal-α-(1,3)-Gal-β-(1,4)-GlcNAc, present on the host epithelial cell membranes [76].

The RBD of TcdA and TcdB consists of 37 or 19 repeats, respectively, building combined repetitive oligopeptide structures (CROPs) [36,77,78,79]. These repeats bind Ca^2+^, thereby enhancing toxin potency [80]. Crystal structures of the RBD have revealed that the short repeats form β-solenoid subunits that pack together in extended rods [81,82,83]. Long repeats also form β-solenoids but are packed differently, yielding kinks in the rods. Each long repeat together with an adjacent short repeat forms a binding site for the saccharide receptors [81] (Figure 1C). Although displaying a narrow and elongated structure, the B domain is fairly rigid, which is consistent with the highly conserved packing interactions and regular rotational relationships observed between the long and short repeats. Furthermore, the B domain extends away from the delivery domain (D domain), so that multiple binding sites are accessible [54,55,78,84].

#### 2.2.3. The Cysteine Protease Domain (C Domain)

The CPD is responsible for the inositol hexakisphosphate (Insp_6_)-dependent proteolytic cleavage of the toxin [38]. This domain is composed of nine-stranded β-sheet flanked by five α-helices. The Insp_6_-binding domain and the active site are located on opposite sites on the central β-sheet and separated by a three-stranded β-hairpin, named β-flap [38]. Several essential catalytic residues, including the putative catalytic triad Asp589-Hys655-Cys700 in TcdA, corresponding to Asp588-His654-Cys699 in TcdB, are conserved [38,60,85,86] (Figure 1C).

Of note, it has been suggested that TcdB may also possess an aspartate protease activity, because the aspartate protease inhibitor 1,2-epoxy-3-*p*-nitrophenoxypropane (EPNP) blocked the processing of TcdB. The Asp1665 residue has been proposed as part of a short Asp-Xxx-Gly motif present in many aspartate proteases [82].

In TcdA, a three-helix bundle region (amino acid residues 767–841 of TcdA) located at the GTD-CPD interface has been identified (Figure 1B). The three-helix bundle is followed by a small globular sub-domain (SGD; amino acid residues 850–1025 of TcdA) and by an elongated hydrophobic helical stretch containing four α-helices (amino acid residues 1026–1135) [34,37] that are part of the D domain (see Section 2.2.4). The role of three-helix bundle is unknown; however, it should be conserved in the two toxins (Figure 1B).

#### 2.2.4. The Hydrophobic Region (the D Domain)

Following receptor binding, the clostridial glucosylating toxins are endocytosed [87]. After endocytosis, the toxins translocate through the early endosomal membrane into the cytosol. This process depends on the acidification of endosomes by vesicular H^+^-ATPase. Bafilomycin, which blocks the H^+^-ATPase, inhibits cytosolic entry of the toxin and intoxication of cells [67]. For TcdB, as the acidity of the endosome increased, a conformational change in the toxin structure happens, with the consequent exposure of the D domain, also defined as the “delivery domain” [88].

The delivery of the GTD domain of TcdB into the host cell cytosol requires the region called the minimal pore-forming region (MPFR), located at the *N*-terminus of the D domain and encompassing amino acid residues 830–990. This region covers only a small part (~40 amino acid residues) of the main hydrophobic region (HR) of TcdB (amino acids 956–1128) [35] (Figure 1B). Of note, the MPFR corresponds to the SGD identified in the HR of TcdA (amino acids 958–1130) [34,36] (Figure 1B,C) (see Section 2.2.3).

The HR of both TcdA and TcdB has been predicted to insert into the endosomal membrane with acidic pH to facilitate the translocation of the GTD into the cytosol [34,35,36,89]. It has been demonstrated that the small region encompassing residues 956–990 of the HR of TcdB is necessary for membrane insertion and pore formation (see Section 3.2) but is not sufficient to translocate the GTD into the host cell cytosol [35]. The translocation region of TcdB encompasses amino acid residues 830–1500 of TcdB [35]. In particular, specific residues located between amino acids 1035 and 1107 have been shown to be crucial for the translocation of TcdB across the endosomal membrane into the cytosol and, consequently, for CDI pathogenesis [89]. The administration of virtually lethal doses of toxins carrying mutations in the translocation region reduces the toxic activity by over 1000-fold and does not induce the death of the mice [89].

The pH-dependent membrane insertion of toxins depends on the presence of two acidic residues (*i.e.*, Glu970 and Glu976) located in a loop connecting two hydrophobic helices crucial for initial membrane interaction and/or insertion. These residues are protonated at the low pH of endosomes. Exchange of these residues into Lys residues caused significant delay and decrease in the efficiency of intoxication as a consequence of the reduced cytotoxicity. Moreover, these mutants exhibited reduced activity to form pores. Notably, change of Glu970 and Glu976 to Ala residues did not block pore formation. This supports the idea that the low pH-triggered conformational changes of the D domain and the neutralization of negative charges in loop regions are both prerequisites of efficient membrane insertion of the pore-forming region [35].

## 3. Mechanisms of Action of TcdA and TcdB

**Figure 2:**
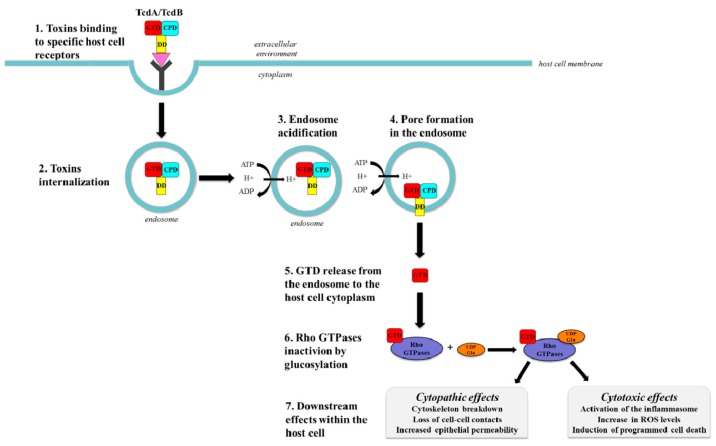
Toxins delivery into the host cell cytosol can be divided into seven main steps: (1) toxin binding to the host cell surface receptor; (2) toxins internalization through a receptor-mediated endocytosis; (3) endosome acidification; (4) pore formation; (5) GTD release from the endosome to the host cell cytoplasm; (6) Rho GTPases inactivation by glucosylation; and (7) downstream effects within the host cell, *i.e.*, toxins-induced cytopathic and cytotoxic effects. For clarity, the color codes used to depict the diverse toxins domains are the same used in Figure 1: GTD: *N*-terminal glucosyltransferase domain (**red**); CPD: cysteine protease domain (cyan); DD: delivery domain (**yellow**).

Toxins delivery into the host cell cytosol can be divided into seven main steps: (1) toxin binding to the host cell surface receptor; (2) toxins internalization through a receptor-mediated endocytosis; (3) endosome acidification; (4) pore formation; (5) GTD release from the endosome to the host cell cytoplasm; (6) Rho GTPases inactivation by glucosylation; and (7) downstream effects within the host cell, *i.e.*, toxins-induced cytopathic and cytotoxic effects (Figure 2).

### 3.1. Toxins Binding to the Cellular Surface and Endocytosis

The intoxication process begins with the endocytic uptake of TcdA and TcdB through a clathrin- and dynamin-dependent mechanism [57,90]. Both toxins enter the cells thanks to the binding of their RBD to one or more receptors present on the surface of target cells [87] (Figure 2).

The two *C. difficile* toxins seem to have different receptors. Sucrase-isomaltase and the glycoprotein 96 (gp96), expressed on human colonocyte apical membranes and in the cytoplasm, have been identified as a TcdA plasma membrane receptor that enhances the cellular entry of the toxin, participates in cellular signaling events and in the inflammatory cascade, and facilitates TcdA cytotoxicity [91,92]. On the contrary, the chondroitin sulfate proteoglycan 4 (CSPG4) and the poliovirus receptor-like 3 (PVRL3) have been identified as specific TcdB receptors, necessary for TcdB-mediated cytotoxicity [59,93].

For a long time, it has been believed that the binding and entry of *C. difficile* toxins into the target cells was mediated only by CROPs. However, recent evidence highlighted that these sequences are not strictly necessary for toxins biological functions [37,61,94]. Of note, TcdA and TcdB lacking the CROP domain are still capable of entering into host cells and causing cell rounding [35,61] thanks to the presence of a functional CROP-independent receptor-binding site, located within amino acids 1349–1811 of TcdB [95]. This domain, which is similar to that present in the TpeL toxin produced by *C. perfrigens*, is required for *C. difficile* toxins uptake into host cells. However, while TpeL enters into the host cells by binding the LDL receptor-related protein 1 (LRP1), the TpeL-like domain of *C. difficile* toxins acts through an LRP1-indendent mechanism [95].

While CROPs are necessary for cellular intoxication since they determine the full potency of the toxins, they are not fully necessary for the cytopathic action [61]. In line with this data, the *N*-terminal GTD domain of TcdB has been shown to interact with target cells [94], and multiple regions, named “modular binding motifs” of TcdB (corresponding to the fragments comprising residues 1372–1848, 1372–1493, and 1493–1848), independently contribute to cellular intoxication [37]. Furthermore, TcdB CROPs do not appear to bind to the two recently identified receptors [59,93], possibly enhancing either in the overall affinity of TcdB for such cellular receptors or having a broader role in the *C. difficile* infection cycle (*i.e.*, enhanced affinity for molecules present on the surface of motile immunological cells, causing the toxin-dependent impairment of cytoskeletal machinery and functionality). Taken together, this evidence may contribute in explaining the historical difficulties in identifying cellular receptors of the toxins, and support a more complex model of clostridial glucosylating toxin uptake than previously suggested [37]. Of note, different cell and species seems to differ in their sensitivity to TcdA and TcdB [37,61].

Once bound to the receptor, the complex toxin-receptor enters the cell through endocytosis and requires an acidified endosome for translocation. Indeed, acidification of the endosome is necessary to alter the toxin’s structure through a pH-induced conformational change most probably between the CROP region and the CPD. In turn, this allows the exposure of hydrophobic regions facilitating the entry into the host cell cytosol [67,88] (Figure 2).

### 3.2. Pore Formation and Translocation

The process that goes from the endosome to the cytosol is called translocation and is guaranteed by a pore-forming mechanism able to penetrate the endosomal membrane and by allowing the release of the toxin into the host cell cytosol [35] (Figure 2). Remarkably, toxins produced by hypervirulent *C. difficile* strains have the ability to enter cells more rapidly and at an earlier stage than historical *C. difficile* strains, due to their ability to undergo acid-induced conformational changes at a much higher pH. Since the pH-induced conformational change is fundamental for toxins membrane insertion and translocation, this data provide evidence for a possible critical change in toxins activity that contributes to the emerging hypervirulence of *C. difficile* [85].

Once in the cytosol, the toxins undergo an autocatalytic cleavage Insp_6_-dependent with the consequent release of the GTD, which finally targets Rho proteins in the cytosol [35,60,82,96,97] (Figure 2).

### 3.3. Rho Proteins Inactivation

TcdA and TcdB, at the end of their autocatalytic process, are released into the host cell cytosol where they glucosylate several members of the Rho subfamily by transferring a glucose moiety from the UDP-glucose to the Thr35/37 residue of Rho proteins. Glucosylation of Rho proteins causes inactivation, thus supporting the notion that the glucosyltranferase activity of *C. difficile* toxins is essential for the disease pathogenesis [32,98,99] (Figure 2).

Rho proteins are GTP-binding proteins belonging to the superfamily of Ras proteins [100,101]. These proteins are located into the cytosol and have multiple functions, such as the regulation of the actin cytoskeleton, of myosin filaments (stress fibers), of cell cycle progression, and of cell division, as well as the regulation of phagocytosis and cytokine production [16,102,103]. The activity of Rho proteins is regulated by a GTPase cycle, in which the GTP/GDP exchange, dependent on the nucleotide exchange factors (GEFs), causes Rho proteins activation. Once activated, Rho proteins interacts with host cells effectors such as kinases and phospholipases that are responsible for the regulation of several signal transduction pathways [16,101].

Among the Rho proteins, those glucosylated the *C. difficile* toxins are RhoA, RhoB, RhoC, RhoG, Rac1, Rac2, Rac3, Cdc42, and TC10 [103]. Glucosylation of Rho proteins inhibits their interaction with the effectors, thus blocking the Rho-dependent cell signaling [104]. Furthermore, glucosylated Rho proteins bind irreversibly cell membranes and prevents translocation of unmodified Rho to the membranes for signaling [105]. Altogether these processes inhibit the interaction with the effectors, thus causing the so-called cytopathic and cytotoxic effects (Figure 2). Notably, differences in the toxin’s substrate specificity lead to different cytopathic and cytotoxic effects [106].

#### 3.3.1. Cytopathic Effects

The cytopathic effects are visualized as drastic morphological changes, such as shrinking and rounding of cells, and initially accompanied by a formation of neurite-like retraction fibers blocking the Rho-dependent signaling and causing the disruption of the actin cytoskeleton and of the tight and adherent junctions, loss of cell-cell contacts, and increased epithelial permeability, all of which are probably the cause of diarrhea [107]. In turn, reduced cell adherence causes apoptosis and cell loss, since epithelial cell renewal is limited and cell proliferation is inhibited as a consequence the inhibition of both cell cycle progression and actin-dependent cytokinesis [16,108,109,110,111,112,113,114] (Figure 2).

#### 3.3.2. Cytotoxic Effects

Besides cytopathic effects, the *C. difficile* toxins can induce cytotoxic effects on the intoxicated cells. The intoxicated cells respond to RhoA inactivation with the upregulation of the pro-apoptotic immediate early gene product RhoB, which transiently escapes glucosylation while being activated and is involved in the regulation of programmed cell death [75,115,116]. The cytotoxic effects are also associated with the activation of the inflammasome by the glycosylated RhoA, which is probably the cause inflammation and colitis induced by *C. difficile* [117] (Figure 2).

##### Induction of the Programmed Cell Death

TcdA and TcdB are able to induce type I (*i.e.*, apoptosis) and type III (*i.e.*, necrosis) programmed cell death [56,118]. To date, it is still controversial whether the cytotoxic effects induced by TcdA and TcdB are dependent on [119,120,121,122,123], or independent of, the glucosyltransferase activity [124].

The crucial role of the TcdA glucosyltransferase activity in apoptosis induction has been highlighted by the fact that TcdA is able to induce apoptosis mainly through the activation of caspase-8 and of cytochrome *c*/caspase-9, in a way dependent on monoglucosylation of Rho protein [119]. This finding was further supported by the observation that TcdA is able to activate caspase-3 and that TcdA mutant characterized by 50-fold reduced glucosyltransferase activity need very higher toxin concentrations to induce apoptosis [123].

TcdB is able to stimulate multiple apoptotic pathways in host cells. Of note, TcdB activities other than glucosylation contribute to cytotoxicity, mainly through caspase-dependent (*i.e.*, caspase-3 activation) and caspase-independent (*i.e.*, Bcl-2-dependent mechanism) apoptotic pathways [125]. Furthermore, it has been demonstrated that TcdB trigger apoptosis via the involvement of mitochondrial ATP-dependent potassium channels. This mechanism is associated with an increased cytosolic calcium concentration and with a mitochondrial membrane hyperpolarization, a state that likely influences the commitment to cell death [126].

With respect to the *C. difficile*-induced necrosis, some studies reported that structurally intact glucosyltransferase-deficient TcdA and TcdB are devoid of glucosylation activity and cytotoxicity [127,128], while other showed that both wild-type and mutant TcdB, having impaired autoprocessing or glucosyltransferase activities, were able to induce necrosis [84,129,130].

##### Activation of the Inflammasome

While glycosylated RhoA is characterized by a “loss of function” phenotype in the regulation of the actin cytoskeleton, it exhibits a “gain of function” phenotype in the activation of the inflammasome. Indeed, TcdA and TcdB are able to increase cytokine production and to stimulate a robust pro-inflammatory response through the activation of pyrin, a sensor of the glycosylated RhoA [131]. Pyrin interacts with the apoptosis-associated speck-like protein (ASC), an adaptor protein that recruits and activates pro-caspase 1 [132]. Caspase-1 is a central regulator of the innate immune defense and activates IL-1β and IL-18. These cytokines cause (among other effects) release of IL-8 and interferon-γ (IFN-γ), respectively. Increase in IL-8 and INF-γ induced by *C. difficile* toxins have been frequently reported [133,134,135,136]. IL-8 is also one of the most potent attractants for neutrophils, which explains the strong neutrophil invasion into colon mucosa in the course of *C. difficile* infection, and is probably responsible for mucosal damage.

Recent findings suggest that the intensity of the host response to infection correlates with disease severity. The pro-inflammatory cytokine interleukin-23 (IL-23) represents a pathogenic mediator during CDI [136,137]. TcdA and TcdB alone are not sufficient for IL-23 production, but synergistically increase the amount of IL-23 produced in response to the MyD88-dependent inflammatory signaling, which includes the pathogen-associated molecular patterns (PAMPs) and host-derived damage-associated molecular patterns (DAMPs) [138]. These signals enhanced the secretion of IL-1, and the subsequent IL-1 receptor signaling is responsible for the increase of IL-23. Notably, IL-1 was increased in the serum of patients with CDI, suggesting that this systemic response could influence downstream production of pathogenic IL-23 [138].

An increasing number of reports have documented the capability of TcdA and TcdB to induce the production of reactive oxygen species (ROS) by target cells. ROS production induced by the toxins, independently of their glycosyltransferase activity, has been suggested [129]. These effects appear to be Rac-dependent since TcdB stimulation caused a transient activation of Rac1 and a robust production of ROS, ultimately causing cell death [129,130].

## 4. Is TcdB the Main Actor of *C. difficile*-Induced Cytotoxicity? Evidence for a Major Pathogenetic Role for TcdB

Initially, in the early rodent models of infection, TcdA was reported to cause intestinal inflammation more efficiently than TcdB [56,139]; however, recently, evidence has increasingly supported the assumption that TcdB plays a key role in the pathogenesis of both localized and systemic CDI. In fact, after the introduction of humanized mice in experiments, the role of TcdB has become more revalued and even overturned, suggesting that the receptors of TcdB could be poorly expressed in several animal models [140].

Already in 1995, Riegler *et al.* tested the effect of purified TcdA and TcdB on human colonic mucosal sheets in a Ussing chamber. Both toxins were able to cause epithelial cell necrosis and electrophysiological changes of the human colon *in vitro*; however, the authors demonstrated that the human colon is 10 times more sensitive to the damaging effects of TcdB [141]. The potency of TcdB has been confirmed by several studies showing that TcdB is 100–10,000 times more potent than TcdA in several cells type [70,142,143].

In 2003, Savidge *et al.* used xenograft in which human fetal intestinal tissue was implanted ectopically into mice [140]. They injected the intestinal graft with a medium containing 40 nmol/L of purified TcdA and TcdB; thereafter, animals were killed after 6 h, and the grafted tissue was removed and analyzed. The authors found that TcdB caused more edema and polymorphonucleocyte infiltration of the mucosa compared to TcdA.

Six years later, Lyras *et al.* tested isogenic *tcdA* and *tcdB* mutants (with disrupted *tcdA* and *tcdB* genes) of a virulent *C. difficile* strain on a hamster model. Hence, *C. difficile* was administered to hamsters and the authors noticed that the disruption of the *tcdB* gene led to a significantly attenuated virulence phenotype, whereas isolates that produced TcdB but not TcdA retained a wild-type virulence phenotype. At the same time, they observed that the presence of TcdA in the absence of TcdB was not lethal in their animal model [144].

An out-of-line experiment that has returned value to the role of TcdA, or more properly to both toxins, was that of Kuehne *et al.* published in 2010. Additionally, these researchers used isogenic toxin mutants of *C. difficile* in which they inactivated either *tcdA*, *tcdB*, or both. They therefore challenged hamsters with *C. difficile* strains after clindamycin exposure. They found that both A^−^B^+^ and A^+^B^−^ mutants were capable of inducing CDI symptoms and ultimately led to animal death after 1.3 days and 4.0 days, respectively [145]. The same authors, four years later, confirmed these data in another hamster model, showing that an isogenic strain producing TcdB alone is more virulent than an isogenic strain producing only TcdA, but the latter is still able to cause disease [146].

In 2013, an animal study assessing the efficacy of specific human monoclonal antibodies against each toxin separately was performed by Steele *et al.* [147]. The animal model consisted of gnotobiotic piglets challenged with 10^7^ spores of *C. difficile* ribotype BI/NAP1/027. Twenty-four hours before the inoculum, six piglets received anti-TcdA antibodies only, six piglets anti-TcdB antibodies only, and six piglets both antibodies. Among the anti-TcdB-only-treated group, no deaths occurred, while among anti-TcdA-only-treated group, 100% developed a systemic disease, and 67% died. In addition, the researchers noticed that the administration of anti-TcdA antibodies, not only failed to protect the piglets but exacerbated the outcome of the disease, leading to more serious consequences, as compared to control animals [147].

More recently, a group of scientists used 3 categories of mutant *C. difficile* isolates: TcdA^−^ TcdB^+^, TcdA^+^ TcdB^−^, and TcdA^−^ TcdB^−^, in 3 different rodent infection models (2 mice and 1 hamster). They used a wild-type TcdA^+^ TcdB^+^ isolate as reference strain. They observed that the survival of animals exposed to TcdA^+^ TcdB^−^ was significantly higher than that of animals exposed to TcdA^−^ TcdB^+^ and TcdA^+^ TcdB^+^ strains. When analyzing extra-intestinal organs, the authors found that mice exposed to TcdA^+^ TcdB^−^ isolates had no damage compared to 80% of those exposed to TcdA^−^ TcdB^+^ and 88% of those exposed to the wild-type TcdA^+^ TcdB^+^ isolate. They observed that, even by increasing the dose of the TcdA^+^ TcdB^−^ strain by 500-fold, no extra-intestinal organ damage was detected [148].

It is also noteworthy that among human infections, cases secondary to strains A+/B^−^ has never been identified, whereas a variable proportion of CDI (depending on geographic location) are caused by A^−^/B+ strains; in fact, clinical isolates from patients with symptomatic CDI invariably produce both TcdA and TcdB or TcdB alone, which suggest that TcdB is the “*conditio sine qua non*” for CDI pathogenesis—at least in humans. In 2002 in Israel, a high prevalence (56.5%) of strains A+/B^−^ from symptomatic patients was observed [149], but the clinical implications of this epidemiological shift are still unknown.

In conclusion, TcdB appears to be the more important toxin from a pathogenetic point of view. Regarding TcdA, there are some differences between the studies, and the reason for these differences is still debated. It has been hypothesized that differences in animal models used, unidentified genetic differences between the bacterial strains used, or in the antibody used could have led to the above-mentioned differences [150].

## 5. Can Toxin-Binding Agents Have a Role in Reducing Pathogenicity?

Given the fact that the main final effectors of *C. difficile* pathogenesis are toxins, and not the bacterium itself, several strategies other than antibiotics have been proposed over the years. The hypothesis of binding *C. difficile* toxins in the intestinal lumen is quite old but still intriguing, and several binding agents have been and are being produced and tested against CDI.

Until July 2015, four *C. difficile* toxin-binding agents were examined in pre-clinical studies: cholestyramine, colestipol, synsorb 90, and tolevamer [151,152]. Particularly, colestipol and tolevamer have been compared with standard CDI treatment in controlled trials. Both resulted in lower microbiological and clinical success compared to the drugs used as control (metronidazole or vancomycin) [153,154].

Recently, Sturino *et al.* investigated the role of calcium aluminosilicate uniform particle size Novasil as binding agent for TcdA and TcdB. The authors conducted an *in vitro* study demonstrating that calcium aluminosilicate removed both TcdA and TcdB at physiologically relevant concentrations. The minimum effective concentration for calcium aluminosilicate was 0.3 mg/mL for TcdA, but 0.5 mg/mL for TcdB, and its affinity for TcdA was greater that for TcdB [155]. There are currently no subsequent studies evaluating this molecule *in vivo*.

Another noble molecule that demonstrated *in vitro* to reduce the mortality of intestinal cells exposed to *C. difficile* TcdB is albumin. Our group tested the effect of human serum albumin (1 × 10^−4^ M) on CaCo-2 cell line exposed to 16 µg/mL of TcdA or TcdB. The results demonstrated that human serum albumin was capable of reducing cell death in the group exposed to TcdB [156]. This effect seems to be attributable to the binding between albumin and *C. difficile* toxins (unpublished data) [157], and an *in vivo* application will soon be attempted.

In conclusion, although luminal binding agents have shown a good potential activity against *C. difficile* toxins, they failed to demonstrate a non-inferiority to standard antibiotic treatments. It is likely that the aim to compare these agents with the standard treatment was too ambitious, whereas their possible use as agents complementary to antibiotics has been not attempted in clinical trials. However, it is possible that this last hypothesis was hampered by pharmacologic interactions existing between toxin-binding agents and antibiotics (e.g., cholestyramine and colestipol bind vancomycin *in vitro*) [151,158]; therefore, they were proposed as alternative and not as adjunctive treatment to antimicrobials.

Other non-antibiotic molecules capable of inhibiting the effects of *C. difficile* toxins have been recently investigated. Human alpha-defensins are proteins isolated from neutrophils and from granules of Paneth cells of the small intestine. These proteins possess a microbicidal potential that derives from their capability to insert themselves (amphipathic molecules) into bacterial membranes, therefore damaging membrane integrity. Alpha-defensins compete with the binding of UDP-glucose to the glucosyltransferase domain of TcdB, thus inhibiting Rho-glucosylation and protecting intestinal cells from TcdB-induced cytotoxicity [159]. In the last two years, other important small molecules capable of contrasting the effects of TcdA and TcdB have been identified and tested both *in vitro* and *in vivo*. Ebselen, a synthetic, low molecular weight, organoselenium compound, is able to block glucosyltransferase release via CPD inhibition, and has been shown to reduce toxin activity ameliorating the disease outcome in a mouse model of CDI [160]. The bile acid derivative methyl cholate, as well as the naturally occurring flavonoid phloretin, has been shown to protect cells from TcdB-induced necrosis acting as an inhibitor of receptor binding and as a non-competitive glucosyltransferase inhibitor, respectively [161].

## 6. Are Severe Clinical Manifestations Associated to the Amount of Toxemia?

*C. difficile* infection can cause systemic complications including ascites, pleural effusion, cardiopulmonary arrest, hepatic abscess, abdominal compartment syndrome, acute respiratory distress syndrome, and renal failure [162,163,164,165,166,167,168]. Additionally, mental status changes have been often reported in complicated CDI cases [169].

In fact, the cellular toxicity induced by TcdA and TcdB is not exclusive to intestinal cells, and as early as 1982, Donta *et al.* demonstrated that the cytotoxicity also occurred in cells derived from mouse adrenal tumors, rat hepatoma, Chinese hamster ovary, and human cervical epithelium [170].

Until recently, it was difficult to investigate the relationship between toxemia and systemic clinical manifestations of CDI because of a lack of sensitivity of detection assays for toxins in blood and/or body fluids. In fact, until April 2015, there was only one report of demonstrated *C. difficile* toxemia [171].

In 2009, He *et al.* published a work where they describe a novel cell-based immunocytotoxicity assay capable of detecting less than 1 pg/mL of TcdA. They tested this assay in fecal and serum specimens of experimentally infected piglets, demonstrating that a low level of toxins disseminated into the circulation of severely affected piglets [172].

Three years later, Steele *et al.* performed a study using the same cytotoxicity assay [173]. They orally inoculated animals with spores of toxigenic *C. difficile* BI/NAP1/027 strains. After the inoculum, toxins were detected in about 30% of animal serums (both piglets and mice), whereas toxins were detected in almost half of the pleuric fluids and two-thirds of the ascitic fluids of piglets and in 100% of the pleuric fluids and two-thirds of the ascitic fluids of mice. Finally, they found a significant association between the presence of toxins in the systemic circulation and the development of systemic manifestations of CDI, in parallel none of the animals with non-systemic CDI had detectable toxin levels [173].

Given these premises, in 2015, the potential of the same cell-based assay for detecting *C. difficile* toxemia on human sera was assessed. Yu *et al.* identified only two toxemia-positive cases among 88 adult patients with CDI. They suspected that specific blockers present in the sera could be responsible for this low detection. Therefore, they depleted the sera from IgG and repeated the test, revealing that, after depletion, the detection limit was by far lower. Therefore, they concluded that, differently from animal models, in humans the detection rate of toxemia is low, likely due to serum anti-toxin antibodies [174]. It is well known that humoral immunity plays a crucial role in protecting from severe and/or recurrent CDI. Patients that acquire *C. difficile* and become asymptomatic carriers have been shown to have higher serum IgG antibody levels against TcdA compared to those who develop symptomatic CDI [175]; in addition, serum IgG levels are lower in patients with relapsing CDI [176], and low anti-TcdA IgG has been reported to be associated with higher mortality rates among CDI patients [177]. The impairment in humoral immunity could partially explain the predisposition to develop CDI in categories such as transplant recipients and HIV-infected patients [8,178]. However, it cannot be completely excluded that detectable toxemia just reflects a deficit in humoral immunity, thus possibly being a surrogate biomarker for a severe course of CDI.

Further studies could possibly find a way to bypass the obstacle represented by the low detection rate of toxemia and to finally define the relationship between the toxemia level and the degree of clinical manifestations in humans.

## 7. Extraintestinal Organ Damage and “Undervalued” Potential Clinical Implications

Unlike other bacterial sepsis, in CDI the systemic involvement is not secondary to the bacterial presence in the bloodstream but rather to the associated toxemia. Until recently, although the pathogenetic process was already known, the phenomenon of extraintestinal damage caused by *C. difficile* toxins has been largely understudied and undervalued. This is likely due to limits in detecting toxemia in humans, because of the lacking capability of current diagnostic tools. Nevertheless, *C. difficile* has been shown to be responsible of systemic complications. In the last five years, animal studies have proven the presence of toxins in several different body fluids, and a recent study has proven toxemia in humans. Systemic complications observed in life-threatening CDI include cardiopulmonary arrest [179], acute respiratory distress syndrome [167], multiple organ failure [180], renal failure [168], and liver damage [165].

Three organs are mainly involved in sepsis: heart, kidneys, and brain. We will focus on studies in which the potential toxin-induced damage to these organs is taken into account, although further studies are required to definitively demonstrate that a direct toxin-mediated involvement occurs and does not result from an extreme sensitivity to the damage induced by toxin levels at detection limit.

### 7.1. Heart

Hamm *et al.* evaluated the effects of TcdB intoxication on a zebrafish embryo model [181]. Zebrafish embryos have the advantage of being transparent so that major organs can be visualized by standard light microscopy. First, the authors demonstrated by fluorescence microscopy the cardiotropism of TcdB in the zebrafish embryo. After 24-h treatment with 37-nM-labeled TcdB (dissolved in sterile embryo water), these toxins localized at the frontal ventral portion of the fish, with specific foci within the pericardial region. Using a TcdB enzymatic domain with a surrogate system composed of *B. anthracis* antigens, the authors were able to demonstrate that the TcdB-induced cardiac damage is not secondary to widespread tissue damage but is related to a specific cardiotropism. Secondly, they demonstrated that TcdB-treated fish experienced a significantly reduced heart rate and a reduction in blood flow as an early sign. At 48 h from toxin exposure, both the atrium and the ventricle appeared deformed, and there was a 60% decline in cardiomyocyte viability. On day 7, 100% of fishes exhibited pericardial edema.

### 7.2. Kidneys

Several studies have shown kidney injury to be associated with CDI. Whether renal failure is a predisposing factor or a consequence of CDI or both is still debated. In the early 1990s, the sensitivity of a monkey’s epithelial culture cells to TcdA and TcdB was demonstrated [182]. In addition, it is known that apoptosis is an important process involved in both acute and chronic renal failure. In 2000, Anderson *et al.* performed a study on cultured renal tubular epithelial cells. They exposed these cells to TcdA and TcdB and found that both toxins were able to inhibit healing of an experimentally induced wound on the monolayers while inducing caspase-dependent apoptosis [183]. TcdA has also been shown to reduce the perfusion pressure and the glomerular filtration rate in rat kidneys [184]; therefore, considering the possibility for an indirect vascular-mediated renal damage. It is clear that this is *in vitro* evidence, and definitive conclusions cannot be drawn without *in vivo* studies.

### 7.3. Brain

There is much evidence that Rho GTPases are important in regulating neuronal survival [185] and that the Rho GTPases inhibition led to cerebellar granule neurons apoptosis. In the last decade, studies demonstrated that global inhibition of Rho GTPases with TcdB-induced cerebellar granule neurons apoptosis through dysregulation of critical prosurvival and proapoptotic signaling cascades [186,187]. In particular, it was observed that TcdB induces downregulation of Rac1 GTPase and Rac-dependent mitogen-activated protein kinase pathway, thus inducing apoptosis in part through reduced degradation of the proapoptotic BH3-only protein Bim. In addition, Rho GTPase inhibition induced by TcdB induces cerebellar granule neurons death through activation of JNK/c-Jun pathway [188]. Some clostridial toxins have been reported to display neurotoxic properties, but an endothelium-mediated damage can be part of the pathogenetic process. Further studies will help to clarify if this *in vitro* evidence could have clinical implications.

## 8. Conclusions

Despite over 80 years of study, *C. difficile*, with its ever-changing epidemiology, still remains a challenging organism to define diagnostically and clinically. While there have been advances in the understanding of the organism’s molecular and genomic mechanisms and in the understanding of the pathogenicity and its host interactions, we are still unable to determine something as simple as whether the fecal presence of this organism equates to disease. In addition, in this era of antimicrobial resistance, there have not been calibrated methods determined so that ongoing patient relapses can be confidently tested as to the antibiotics efficacy. The search for non-antibiotic therapies such as recolonization of an infected gut by donor fecal material has shown promising outcomes. What is emerging is that the scope of clinical manifestation has moved beyond the gut to other organs such as the heart, kidneys, and brain, indicating systemic toxemia is occurring and possibly plays a fundamental role in determining the prognosis of CDI patients. Up to now, there have not been any reliable methods to measure toxin load in serum or tissue in humans. It appears that, for every advance we make in understanding this organism and its host interaction, new questions arise.
